# Early Implementation and Evaluation of StepUp for Dementia Research: An Australia-Wide Dementia Research Participation and Public Engagement Platform

**DOI:** 10.3390/ijerph182111353

**Published:** 2021-10-28

**Authors:** Yun-Hee Jeon, Mirim Shin, Adam Smith, Elizabeth Beattie, Henry Brodaty, Dennis Frost, Anthony Hobbs, Piers Kotting, Glenys Petrie, Martin Rossor, Jane Thompson, James Vickers, Donna Waters

**Affiliations:** 1 Susan Wakil School of Nursing and Midwifery, The University of Sydney, Sydney 2006, Australia; mirim.shin@sydney.edu.au (M.S.); donna.waters@sydney.edu.au (D.W.); 2 Office of the National Director for Dementia Research, National Institute for Health Research (NIHR), University College London, London WC1N 3BG, UK; adam.smith@nihr.ac.uk (A.S.); m.rossor@ucl.ac.uk (M.R.); 3 School of Nursing, Queensland University of Technology, Brisbane 4059, Australia; elizabeth.beattie@qut.edu.au; 4 Centre for Healthy Brain Ageing (CHeBA), University of New South Wales, Sydney 2052, Australia; h.brodaty@unsw.edu.au; 5 StepUp for Dementia Research, The University of Sydney, Sydney 2006, Australia; dennis.frost@bigpond.com (D.F.); glenys.p@hotmail.com (G.P.); jantho@bigpond.com (J.T.); 6 Little Company of Mary Health Care Ltd., Canberra 2617, Australia; Anthony.Hobbs@calvarycare.org.au; 7 College of Medicine and Health, University of Exeter, Exeter EX1 2LU, UK; P.Kotting@exeter.ac.uk; 8 Wicking Dementia Research & Education Centre, University of Tasmania, Hobart 7001, Australia; james.vickers@utas.edu.au

**Keywords:** dementia, research participation, recruitment, registry, digital platform, consumer involvement, consumer engagement

## Abstract

Recruiting participants for dementia research takes time. For those who are interested, opportunities to participate can be *ad hoc*. Delays in finding the right participants can result in studies taking longer to deliver, often requiring funding extensions, and ultimately increasing the cost and limiting the effectiveness of research and evaluation. To address these issues, a digital platform, StepUp for Dementia Research, was developed in 2019 and evaluated through ongoing data analytics, researcher feedback and annual volunteer surveys in 2019 and 2021. Using innovative matching technology, StepUp provides volunteers with an opt-in, secure way of registering interest in dementia studies and allows researchers to access matched volunteers in Australia. As of June 2021, 1070 volunteers registered (78% female), and 25 organizations became ‘champions’ for StepUp promotion. Of 122 registered researchers, 90 completed training. Forty studies from 17 research/health institutions recruited participants using StepUp. The evaluation demonstrated program feasibility and recruitment efficiency with a high level of satisfaction from users. Evaluation outcomes highlighted disparities in public participation in dementia research (e.g., gender, education and race/ethnicity) and provided valuable insights for further enhancements of StepUp. A concerted and strategic effort is needed by leading registries such as StepUp to ensure narrowing volunteer participation gaps in dementia research.

## 1. Introduction

Globally, an estimated 55.2 million people have dementia [1]. Despite significant medical and technological advances, and decades of research to find a cure for dementia, dementia remains largely incurable. Timely, accurate diagnosis and early intervention are critical to help people with dementia and their families to access appropriate services and treatments. Moreover, many are experiencing significant challenges associated with delayed diagnosis and limited access to the care and support services they need [2]. Research evidence is a building block of good practice and service delivery; ensuring service and care quality requires robust evaluation research. The WHO Global Action Plan on the Public Health Response to Dementia 2017–25 clearly articulates the importance of improving dementia research (Action 7) and the crucial role research plays in all other action areas [2]. Effective recruitment of research participants is a key element in determining the success of research outcomes and outputs, but recruitment processes can be highly costly and time-consuming.

For example, 40% of the US pharma/biotech research budget is spent on clinical trials (US$7 billion) and the cost of recruitment is estimated to be US$1.89 billion per annum. Over two thirds of trial sites fail to meet their enrolment target numbers and up to 50% of sites have one or no participants enrolled in their studies [3]. A plethora of papers document problems associated with the failure of timely recruitment for research and its economic consequences [3,4,5]. Multiple reasons exist for the common challenges the public/patients face in terms of their lack of awareness and understanding about available research studies and the meaning and implication of research participation, which in turn generates uncertainty, fear, and concerns about benefits, risks, and privacy [3,4,6]. Delays in finding the right research participants who meet eligibility criteria can result in studies taking longer to deliver, often requiring funding extensions and ultimately, limiting the effectiveness of research and evaluation [3,5].

Recruiting participants into dementia research is even more complex because of stigma, low dementia literacy in the community and the therapeutic nihilism towards dementia that prevails in our society [3], the requirement for participants to have a study partner (e.g., caregiver), the multimorbidity present in most older participants that may disqualify their study eligibility and, for some studies, older people’s lack of internet access and technological fluency [4,6,7]. Despite growing evidence for the prevention (or risk reduction) of dementia [8,9,10], there is a continued lack of knowledge and understanding, with a widespread public misconception that dementia is a normal part or non-preventable consequence of aging [11,12]. Commonly recommended approaches and strategies to improve trial recruitment include enhancing awareness about research participation opportunities (e.g., advertisement, mailing lists and education of health professionals) and outreach (e.g., boosting clinical referrals, satellite clinics and community participatory action research, especially for targeting minority groups) [7,13].

In the past decade many countries have demonstrated government-level commitment to boosting research funding for dementia [5,14,15]. However, recruitment challenge has not been adequately or systematically addressed and in fact the challenge continues to grow. Fargo et al. described this as “the crisis in participant enrolment, a problem that is poised to derail the first goal of the National Plan to Address Alzheimer’s Disease—to prevent and effectively treat Alzheimer’s disease by 2025” (p. 1113) [5]. Participant/volunteer recruitment registries have been increasingly popular in this regard [6,13,16]. Unlike the traditional prospective recruitment processes of advertising study details and/or seeking referrals from health services/clinicians, volunteer recruitment registries provide systematic mechanisms for consenting volunteers to be contacted either by researchers directly (e.g., UK’s Join Dementia Research and StepUp for Dementia Research in Australia) or by the registry staff. Examples of the latter are, the Alzheimer’s Association’s TrialMatch [17], the Alzheimer’s Prevention Registry [18] and Gene Match [19] in the US, and the Dutch Brain Research Registry [20]). Upon contact and further screening, willing and interested volunteers are given the opportunity to provide informed consent to the specific study. Over the past ten years volunteer recruitment registries have shown promising outcomes in tackling the dementia specific research recruitment challenge [4,20]. Additionally, they provide a mechanism to support continuation of study recruitment when face-to-face physical services are suspended or impacted by closures, as seen during the COVID-19 pandemic.

Dementia is the second leading cause of death of Australians, and the single greatest cause of disability in older Australians [21]. The latest estimate for dementia prevalence in Australia is 472,000 people [22], a figure that is expected to triple, reaching over one million by 2058 [22]. As in many other countries, Australians living with dementia and those interested in dementia research have *ad hoc* opportunities to participate in research. Local communities, interest groups and online services, provide limited support for the public to participate in studies they hear about in the media. To addresses these issues and challenges in dementia research in Australia, StepUp for Dementia Research (StepUp hereafter) was developed and piloted between 2018 and 2020. StepUp aims to empower the public/patients and encourage broader community engagement in research and improve opportunities for access to research studies by facilitating recruitment efficiency, thereby reducing the cost and time to achieve study results. StepUp was modelled on the highly successful ‘Join Dementia Research (JDR)’ service, established in the UK in 2015. JDR has demonstrated benefits in terms of increased research recruitment efficiency, access to research for the public and for researchers, public engagement, and attitudinal change in dementia and research participation [23]. Since its inception in early 2015, UK JDR has signed up over 50,000 Volunteers and enrolled over 33,000 individuals into over 300 dementia studies. Over 1750 researchers are registered users, spanning over 296 National Health Services, university, and commercial research sites [24]. A successful collaboration between the two countries meant that the Australian team could leverage the investment made by the UK Government by using the knowhow of the JDR platform as the basis for StepUp and incorporating a range of improvements and innovations to the current UK JDR.

In this paper the key processes and outcomes of StepUp’s early implementation are described. Firstly, the paper focuses on the feasibility of StepUp in New South Wales (NSW) and Western Australia (WA), which was followed by a nationwide roll-out. Key milestones for volunteers and registered studies in StepUp are examined. Secondly, it assesses how well the early implementation achieved the intended goals, by exploring the experiences and perceptions of volunteers and researchers registered in StepUp. Key gaps in implementation are discussed to help further improve StepUp in terms of participant diversity and inclusiveness.

## 2. Materials and Methods

### What Is StepUp for Dementia Research and How does it Work?

**Figure 1:**
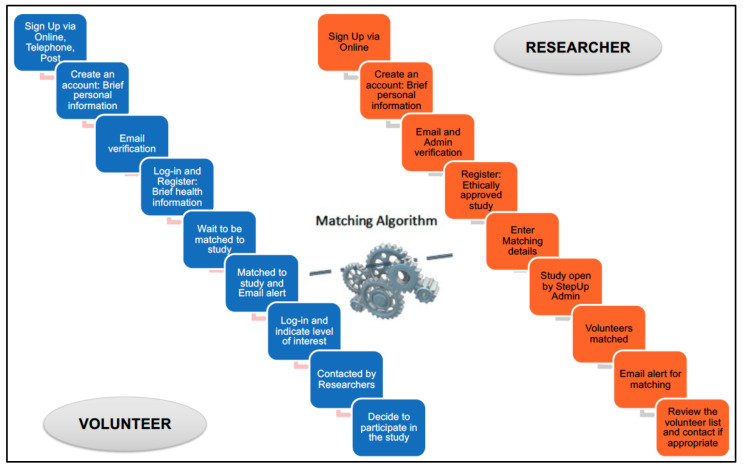
Registration and matching processes.

StepUp is an online, telephone and postal program that uses innovative matching technology to provide people with an opt-in, secure way of registering interest in studies, and allowing researchers to access directly matched volunteers, to speed up study delivery. The platform consists of three key interfaces: volunteer registration, researcher/study registration and matching between volunteers and studies. Figure 1 illustrates the processes involved in registration and matching. StepUp promotes ethically approved research and is accessible to anyone over the age of 18 years who is interested in taking part in dementia research. It is an Australia-wide ‘one stop shop’, public engagement and dementia research participation platform, designed to improve community participation and dementia literacy and research efficiency. It connects adult individuals (both with and without dementia) with researchers delivering studies into prevention, diagnosis, treatment, and care in dementia. For those who have limited or no capacity to register by themselves, the program offers options for assisted registration by a family or friend and proxy registration by a guardian, attorney or authorised person on their behalf. Once registered, volunteers will be asked to login annually to check and update their record. If they fail to update their record for more than 3 years their records will no longer exist. Participants are free to withdraw from the service at any time.

**Figure 2:**
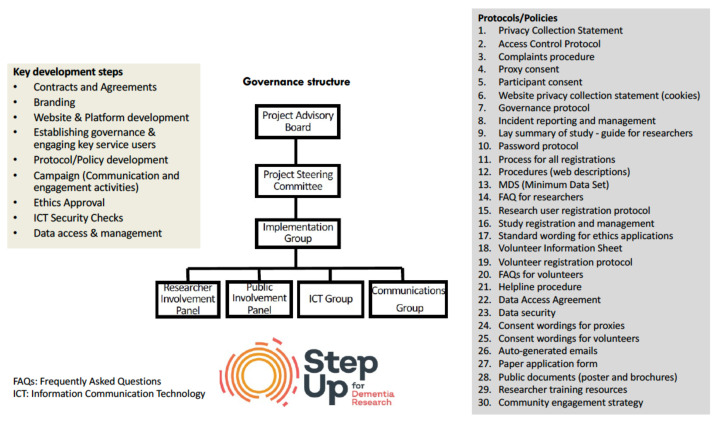
StepUp for Dementia Research development, governance and protocols.

As part of StepUp’s implementation strategies, a Community Engagement Strategy protocol was developed outlining key communication and campaign strategies targeting dementia and aged care peak bodies, health networks and memory clinics, professional bodies, and research networks. Community engagement strategies include direct electronic and postal communications, presentations and attendance at local, national, and international conferences and forums, use of media including TV, radio, online news, and social media. The StepUp team has also engaged with key influencers and organizations through the StepUp ambassadors and organization champions program to raise awareness and promote StepUp. StepUp implementation is fully guided by a governance structure and 30 protocols, and ethics and cybersecurity guidelines. Figure 2 outlines key steps in the development and implementation of StepUp, its governance and protocols.

The early implementation of StepUp was evaluated against the following performance indicators:

(1) platform functionality and feasibility (i.e., effective website and registration processes via online, helpdesk and post, and deliverables including ethics approval, data management and cybersecurity);
(2) registration reach (with initial focus on NSW and WA, followed by a national rollout) and the number of volunteers, researchers and studies; and
(3) user experience (i.e., support for volunteers, researcher training and support, and institutional agreements).

Data collection directly from the StepUp platform included demographics, numbers signed up via the helpdesk/website/paper form, number participating/enrolled in research projects, and withdrawals, surveys of volunteers registered in StepUp, feedback from researchers, and brief case analyses of research studies that used StepUp for recruitment. Two online/paper surveys, in mid-November 2019 and February 2021, were conducted with StepUp volunteers, with the invitation sent to all volunteers registered in StepUp. Descriptive statistics were used to analyze all quantitative data.

## 3. Results

### 3.1. Platform Functionality and Feasibility

The volunteer and researcher registration functionality of StepUp was successfully launched in June 2019 and study registration and matching functionality was fully commenced by September 2019. These two staged functionality releases were deliberate to ensure there were enough volunteers and registered and trained researchers before StepUp offered the matching service for recruitment. During the COVID-19 pandemic, StepUp was able to continue the service to support studies for recruitment, and in some cases, was the only source of recruitment during the pandemic.

### 3.2. Reach and Characteristics of Volunteers and Researchers Registered in StepUp

As of June 2021, StepUp had registered 1069 volunteers who could be matched to research studies, 95% of these had joined via online and 42% were from NSW. Most of the registered volunteers were either 45–64 years of age or in the 65+ age groups (41% each group). Approximately 5% of the volunteers were registered by proxy or with assistance from a relative. Nine per cent of volunteers were diagnosed as having a form of dementia and 2% with Mild Cognitive Impairment (MCI). Of these, approximately 93% described their symptoms as mild to moderate. Forty-nine per cent of all volunteers had a first-degree family history of dementia and 19% had some form of memory or possible dementia related problem. Twenty-three per cent of the volunteers were currently supporting/caring for a relative/friend/spouse/partner with dementia. In terms of the volunteers’ ancestry, 66% selected Australian, 32% English, 14% Irish, 12% Scottish, 2% Chinese while only seven volunteers identified themselves as Aboriginal or Torres Strait Islanders. Almost all (99%) of 780 volunteers who answered non-mandatory questions for registration (73%) selected English as their preferred language; only 15 (<2%) chose other languages.

Many of the 1069 volunteers indicated that they signed up to StepUp because they had heard about it through dementia advocacy organizations (28%) and social media and internet searching (19%). Other sources were mainstream media (14%), word of mouth (12%), health/aged care services (5%), and public events/conferences (4%). Between September 2019 and June 2021, 122 researchers across Australia signed up and created researcher accounts (NSW 63%, QLD 12%, VIC 12%, Other 14%); 90 of these completed mandatory one-hour online training. These researchers were from 17 universities/research institutions and health service organizations that approved Data Access Agreements, a gateway for their researchers to access StepUp.

### 3.3. Key Milestones for Volunteers and Registered Studies in StepUp for Dementia Research

A key milestone for the StepUp service is the number of volunteers who had been matched to registered research projects and enrolled as participants. For online surveys that did not require direct researcher contact or enrollment, 1085 volunteers had been matched to at least one study (many were matched to more than one study). For other studies that required direct researcher contact, researchers were alerted about matched volunteers online and consequently required to change the matched volunteers’ status as they progressed (from *Matched* to *Contacted, Screening, Declined, Enrolled, Ineligible, Completed*). As of June 2021, 40 studies (9 intervention and 31 non-intervention studies) had recruited volunteers using StepUp (16 studies closed and 24 were still recruiting). Twenty-two studies had been conducted by NSW researchers and ten by researchers from other states. Of the 40 studies, 14 targeted people living with dementia, eight studies were for people with MCI, 17 involved carers, and eight studies recruited people with memory concerns. Eleven studies were looking for healthy volunteers. Two of the studies required an online survey for all StepUp Program registrants. Table 1 provides case analyses of individual studies that have used StepUp for recruitment including the time taken for volunteers to be matched to a study upon enrollment.

### 3.4. The Experience of Volunteers and Researchers Registered in StepUp for Dementia Research

#### 3.4.1. Characteristics of Volunteers who Responded to the Surveys

As shown in Table 2, the demographic characteristics of 769 respondents from both survey time points (*n* = 319 for 2019; *n* = 450 for 2021) are very similar. Most volunteers were aged 45–64 years or 65+ years, reflective of current volunteer data. Most survey respondents were female (80%) and English speaking (99%). Almost 70% were born in Australia, while 16% were from the UK. Education levels were relatively high, with more than 60% of survey respondents having completed a university degree (bachelor’s degree or higher education). Approximately 50% of the volunteer respondents were retirees, and about 40% worked either full-time or part-time. Two-thirds of the volunteers reported that they were married or in a de facto relationship.

Approximately 40% of respondents were either currently, or had previously, cared for someone with dementia. One in three respondents had a relative or a friend with dementia and 3% identified as living with dementia. More than 80% of the respondents registered themselves as a volunteer, 3% of volunteers registered on behalf of someone else and 15% registered as a volunteer as well as a proxy (both 2019 and 2021 surveys).

In 2019, 66% of respondents (*n* = 199) had visited the StepUp website between 1–5 times and 1% (*n* = 4) had visited more than 10 times. Thirty per cent of respondents (*n* = 90) never visited the StepUp website after registration. In 2021, 73% (*n* = 300) had visited the StepUp website occasionally, especially when they received email or a newsletter from StepUp. However, only 1% (*n* = 5) were frequent visitors to the website, and 26% (*n* = 107) still never visited the website.

#### 3.4.2. Volunteers’ Experience with StepUp Registration: Online, Helpdesk and Post

**Figure 3:**
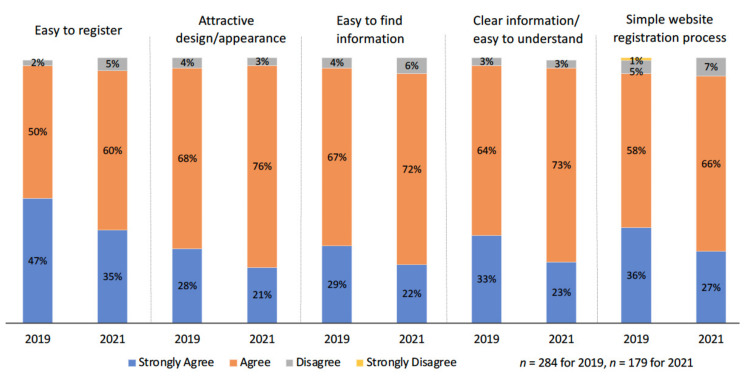
Experience with registration processes 1. Note: 2021 survey respondents (*n* = 179) were newly registered since 2019 survey.

Approximately 90% of survey respondents registered with StepUp via the website (*n* = 284 for 2019, and *n* = 406 for 2021). Generally, people had a positive experience with online registration (Figure 3), with over 93% of the respondents *Agreeing* or *Strongly Agreeing* that it was easy to register and find information, and had an attractive design/appearance, clear information and a simple website registration process. However, the proportion of people who *Strongly agreed* in 2019 was reduced across all these questions in 2021 (Figure 3). All fifteen 2019 survey respondents who registered with StepUp via the helpdesk *Strongly agreed* or *Agreed* to ease of registration via the helpdesk, usefulness of helpdesk information and operator’s ability to answer questions. In addition, two respondents who registered using a paper form, *Strongly agreed* and *Agreed* to these aspects of registration.

**Figure 4:**
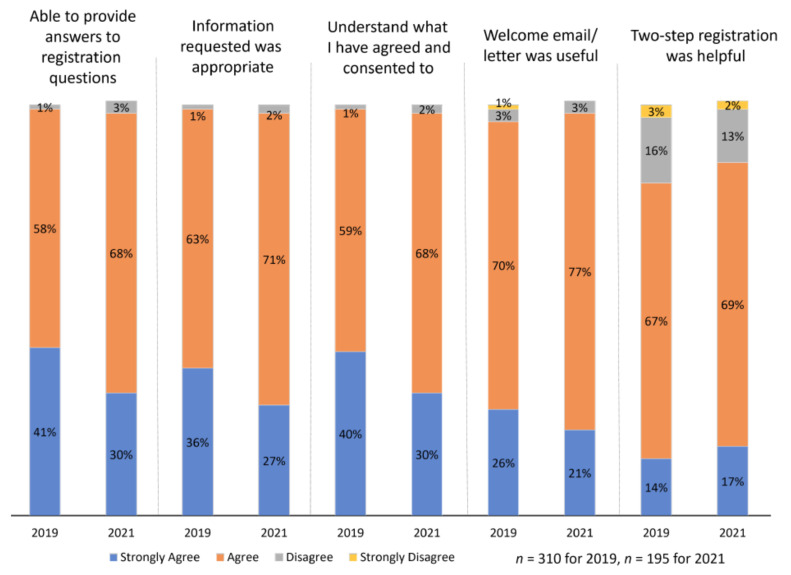
Experience with registration processes 2. Note: 2021 survey respondents (*n* = 195) were newly registered since 2019 survey.

As shown in Figure 4, a total of 505 respondents from both surveys completed a question regarding the overall registration process. They generally *Agreed* or *Strongly agreed* that the registration question and information was appropriate, they understood what they consented to and that the welcome email/letter was useful. However, 19% of 2019 respondents (*n* = 58), and 15% of 2021 respondents (*n* = 27), did not find that the two-step registration was helpful.

Twenty-seven respondents provided suggestions to improve the registration process (*n* = 22 for 2019 and *n* = 5 for 2021). Suggestions included a simpler registration process and passwords policy, and clear wording during the registration. Other comments related to minor website design issues and the need for more advertising so that more people could join StepUp.

#### 3.4.3. Volunteers’ Motivation to Join StepUp and Interest in Broad Research Activities

StepUp provides no monetary incentive. When asked about their motivation for joining StepUp (response *n* = 319 in 2019, and *n* = 450 in 2021) most respondents selected benefits to mankind such as improved dementia diagnosis, prevention, treatment, and care (81% for 2019; 82% for 2021), and wanting to help researchers (61% for both 2019 and 2021) as the two main motivations. Other common motivators included personal benefits, learning more about dementia and being affected by dementia directly or indirectly (38–47%), or as something interesting to do (18–20%).

Over half of respondents (51% in 2019; 53% in 2021) stated they were aware of dementia research in Australia before registering in StepUp, while only a third (33% in 2019; 34% in 2021) said they knew how to volunteer to participate in dementia research. In the 2021 survey, 80% of respondents (*n* = 334) indicated that StepUp had increased their awareness of dementia research and 76% (*n* = 319) *Agreed* that StepUp had made it easier to participate in studies, a slight increase compared with the 2019 survey.

**Figure 5:**
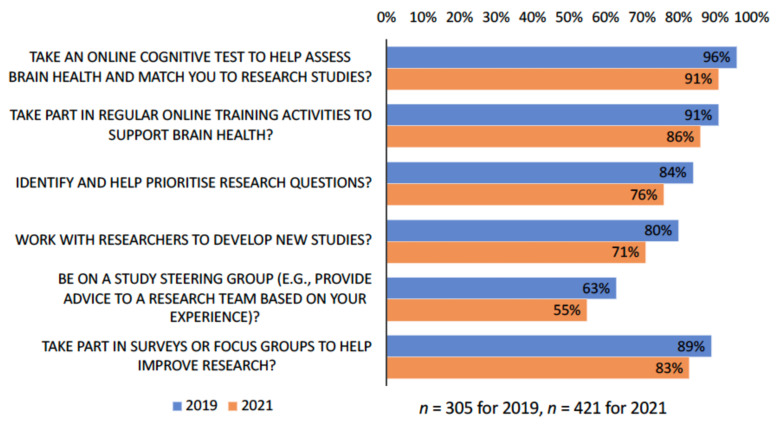
Involvement in research.

When asked of their willingness to be involved in research in other ways, beyond being a research subject/participant, a substantially large proportion of respondents to both surveys (Figure 5) expressed strong interest in participating in online cognitive tests or cognitive training (over 86–96%), or in public involvement in research. A smaller proportion of the respondents showed interest in being on a study steering committee.

#### 3.4.4. Volunteers’ Experience with Research Participation through StepUp

In the 2019 survey, 23% of 303 respondents (*n* = 70) indicated that they had been matched to at least one study, while 62% (*n* = 187) said they had not been matched. Of the 70 respondents who had been matched to a study, 86% (*n* = 60) found the study information was easy to understand. Of 40 people who were contacted by the research team (not all matched would be contacted by researchers), 87.5% (*n* = 35) said the information provided by researchers helped them to decide whether or not to participate (23 subsequently participated in the study), and 90% (*n* = 36) were satisfied with their interaction with researchers.

The 2021 survey responses indicated improvements in volunteer experience with research participation and StepUp services. Of 430 respondents, more than half (*n* = 231) indicated that they had been matched to a study, with 94% (*n* = 217) stating that the study information was easy to understand and 74% (*n* = 171) had been contacted by the research team for the matched studies. Among those who were contacted by a researcher, 93% (*n* = 159) said that the information provided by the researcher helped them to decide their participation (135 enrolled in the study afterwards). Eighty-nine percent (*n* = 152) of respondents were satisfied with the interaction with the research team. Notably, 17 said that the study was delayed, or they could not participate in the study due to COVID-19.

About 70% of survey participants found the StepUp Program *Good* to *Excellent* in their overall experience with research participation (68% for 2019 and 71% for 2021). In both surveys, the main reasons for dissatisfaction related to not being matched to any studies, or not being eligible for matched studies. A small number of respondents had some difficulty using the website. In both surveys, nearly 90% of volunteers said they would recommend StepUp to their friends, colleagues or family members.

#### 3.4.5. Researcher Experience with StepUp

A small number of researchers provided written feedback on StepUp (n = 12) over the course of the implementation. Overall, most researchers were very satisfied and extremely likely to recommend StepUp to others and to use it for their future studies. They were satisfied with the study registration processes and functionality (e.g., matching criteria, volunteer identity protection and researcher training), but two researchers would have liked more control over the registration processes and management, with less time taken to obtain the Data Access Agreement from their organization (a mandatory requirement for the use of StepUp, designed to protect volunteer privacy). Examples of the comments include: *“StepUp is an incredible service. Every part of the registration process was well-explained and easy to follow on. The StepUp administration team is professional and able to answer all queries.”*

As shown in Table 1, about 50–60% of online survey respondents were successfully recruited via StepUp. While researchers often used additional methods of recruitment such as hospital noticeboards, an institutional registry, advertising with community groups and word of mouth, their feedback was clear that StepUp accelerated the recruitment process. For example,

*“StepUp contributed more than half of our responses! We were thrilled by the number of responses we received though StepUp.”*

*“The survey has done really well since being on Step-up—it’s such a brilliant resource for age-related research!”*

Of 17 observational studies registered, the mean recruitment rate via StepUp was between 35–100% (Table 1). Online surveys and interview studies showed most success in recruiting participants using StepUp. For example, a study investigating carers’ experience of medication management advice for people with dementia aimed to interview 50 carers of people living with dementia and started recruitment of participants through professional networks, consumer advocacy groups (e.g., Dementia Australia, COTA, Cognitive Decline Partnership Centre, Carers Australia), and media outlets (newspaper and social media), as well as StepUp. Twenty-two volunteers (>73%) were enrolled via StepUp within three months compared with eight volunteers from all other sources over a six-month period.

Low recruitment success rates were sometimes attributed to factors beyond the control of StepUp. One study could not proceed due to a drug supply issue and another research team had to withdraw their study from StepUp due to the distance volunteers were required to travel. A researcher from one study that had the lowest recruitment rate (35%) provided this comment:

*I think what influenced our relatively low number of StepUp volunteers is that we began recruitment in November and conducted this research over Dec/Jan so it might have corresponded with holiday time when many people might not have been available to participate in research. The platform was easy to use and to navigate. Volunteers were often responsive to invites for participation and all volunteers were interested in the topic of our research and provided some great feedback which was helpful.*

## 4. Discussion

StepUp is the first digital public/researcher matching in Australia designed to facilitate recruitment efficiency and broader community engagement in dementia research. Our evaluation of the early implementation of StepUp has shown that it is not only feasible as an effective recruitment service for researchers, but the model also has further potential for application to the wider community in a short timeframe. Within two years of commencing the matching service for dementia research, StepUp was able to secure over 1000 full registrants (volunteers), predominantly via online (95%), 25 organization champions, and over 120 researcher registrants from 17 universities/research institutions across the nation (there are 43 universities in Australia), leading to the support of over 40 dementia research studies. Two volunteer surveys about the performance of StepUp have shown consistently high satisfaction with the registration processes and support services, and researcher satisfaction with the service has also been high.

Data analytics confirm some of the typical population characteristics seen in dementia research but also suggest areas where representation is low. For example, StepUp volunteer profiles consist of a high proportion of females (over 75%), over 80% of volunteers are aged 45 years and over, almost half of the volunteers have a first-degree family history of dementia, a quarter of the volunteers are in a caring role, about 20% with subjective memory concerns, and only 11% of volunteers are diagnosed with dementia or MCI. These figures indicate key areas to improve StepUp’s ability to provide representative samples by concerted efforts to engage more male volunteers and more people living with dementia and MCI. These challenges are not unique to StepUp. Other well-established internet-based recruitment registries such as GeneMatch [19] and Brain Health Registry [25] in the US have shown a similar trend. Findings from the UK JDR also indicate that initially, most of their volunteers were female (72%) and less than 11.5% of their total volunteers had a formal diagnosis of dementia or MCI [26]. However, since the changes to their promotional strategies, in particular targeting diagnosticians, clinics and hospitals, the proportion of volunteers with a confirmed dementia diagnosis in the JDR databank has risen to 26% in 2020/21 [27]. Little is known about reasons for female dominance in research volunteerism. A study of gender differences in volunteering and charitable giving showed that females were more motivated to help others, which was associated with higher scores on measures of agreeableness, subjective religiosity, moral obligation and prosocial role identity, than their male counterparts [28]. Nevertheless, there is no research addressing the question of why men are less likely to volunteer for research participation, a topic that is difficult to investigate as those males not participating in research cannot be asked.

The StepUp registration process does not capture the volunteer’s educational background and therefore the study is unable to draw a direct conclusion that StepUp volunteers represent a higher educational background than the general population. However, some association can be made, signaling the need to reach out to those people who are less familiar with dementia research and possess a lower educational background. The survey findings suggest that volunteers’ experience with research participation through StepUp has improved over time, possibly due to the fact that more people have had an opportunity to participate in studies through StepUp for recruitment. In terms of volunteer awareness of participating in dementia research, similar observations have been found in both StepUp and JDR. The volunteers of both services report raised awareness of dementia research after registration (80% in StepUp; 81% in JDR) and improved access to participation in research studies (76% in StepUp and 73% in JDR) [26,29]. However, the survey findings suggest that StepUp volunteers are more familiar with dementia research than JDR volunteers: over 50% of StepUp survey respondents had some level of dementia research knowledge before registration, and about one third of the survey respondents were aware of how to volunteer to participate in dementia research prior to StepUp, compared with less than 15% of JDR volunteers with the same answer. These findings may be a reflection that over 60% of the StepUp survey respondents have a university or higher degree.

In addition to possible education disparities, an evaluation suggests that StepUp has not adequately addressed the issue of racial/ethnic disparity. Australia is a multicultural society. Almost half of all Australians (10.6 million) are either born overseas (6.2 million) or had one or both parents who were born overseas (4.5 million). Among people aged 65 years and over, 37% are born overseas and 12% speak a language other than English at home, with Italian and Greek being the most commonly spoken languages other than English [30]. However, most StepUp volunteers are Caucasians (90%) and speak English as a primary language (99%), which does not reflect the nation’s diversity. The issue of under-representation or under-reporting of people with culturally and linguistically diverse (CALD) backgrounds in dementia research has long been raised in Australia and internationally [31,32,33]. People from CALD backgrounds face numerous barriers when it comes to accessing services due to difficulties with language, their lack of knowledge of the services, lower health literacy, and limited culturally and linguistically appropriate services and assessment, all of which become a major impediment to the accurate diagnosis and treatment and care for people with dementia. Dementia further presents unique challenges to people from non-English speaking backgrounds, often causing them to revert to their primary language. Furthermore, there is a lack of understanding of dementia by many people in CALD communities [31,32,33].

StepUp has only seven volunteers identifying as Aboriginal or Torres Strait Islanders. There were an estimated 787,000 Aboriginal and Torres Strait Islander Australians in 2016, representing 3.3% of the total Australian population [34,35]. As a group, First Nations people experience widespread disadvantage and health inequality, including higher rates of dementia. Aboriginal and Torres Strait Islander people experience dementia at an earlier age and at a rate 3 to 5 times higher than the general Australian population [36]. Despite these statistics, dementia in Aboriginal and Torres Strait Islander communities is often not well understood or recognized by health workers and service providers. Geographical constraints in the provision of services, a lack of education and awareness in communities and by health workers, and the prevalence of other chronic diseases have all posed considerable barriers to the recognition of dementia as an emerging health issue [36].

The StepUp Community Engagement Strategy identifies the importance of diversity and inclusiveness and continues to reach out to CALD and Aboriginal and Torres Strait Islander communities using community talks, media and social media outlets. What has become clear is that the complexities in engagement are far deeper than previously understood and StepUp’s approach has not been sufficient to reach these important communities. Racial/ethnic disparity in dementia research participation is a well-known phenomenon, albeit not unique to dementia fields. This issue has been highlighted in other internet-based recruitment registries such as GeneMatch [19] and Brain Health Registry [25]. Despite a steadily growing number of dementia research studies trying to address this disparity, there appears to be no easy solution to tackle this problem, as successful models are often not transferable outside specific localities or projects [13,19]. A particular race/ethnicity specific registry may be a solution as has been used for CARE (https://careregistry.ucsf.edu (accessed on 26 October 2021), which is designed to address a lack of engagement among Asians, Asian Americans and/or Pacific Islanders in research concerning Alzheimer’s disease and related dementias (ADRD), aging, and caregiving related research. However, the same issue as to how to make the wider shift to attract people of diverse backgrounds to sign up remains to be resolved. Other studies have identified barriers to inclusion such as fear and a lack of trust, often originating from a lack of knowledge and understanding about what it means to consent to participate in research, as well as past negative and/or historical events experienced by some minority groups [37,38,39,40]. Therefore, a solution may require firstly building a trusting relationship between researchers and communities, and the use of community-based participatory research [13]. Others also propose that grant bodies provide proactive guidance to researchers for example, making it a condition of funding to address diversity issues, and investment of additional funding to support translation/interpreter services [31].

StepUp’s primary target audience is people who are interested in contributing to society and/or enhancing knowledge through participation in research into the understanding of dementia, its prevention, diagnosis, care, treatment, and service options. StepUp aims to provide a unique opportunity for those living with dementia, their carers and families and the wider public to make a positive and recognized contribution to the community at large through participating in research. What is striking in these surveys is that a substantially high proportion of people expressed interest not only in being a research subject/participant but in being involved in research and collaborating with researchers in other ways. The findings echo a steadily growing interest in ways of involving and engaging the public in research and the notion of co-production, partnership and participatory research in scientific communities (i.e., consumer/community involvement in research, citizen science, patient/public involvement in research, co-design research) [41,42,43]. The WHO’s Global status report on the public health response to dementia further recommends a concerted effort to improve not only the extent of public involvement in dementia research but also the diversity and inclusiveness of various socio-economic and ethnic groups within countries [44]. This trend signals a new opportunity for registries such as StepUp to embrace, promote and support the interests of the public in engaging with and being involved in research beyond being participants. Our evaluation findings also suggest that with enhanced function, StepUp has the potential to improve dementia literacy and reduce stigma towards dementia and inform and guide dementia services and research policy development.

## 5. Conclusions

StepUp is not a time limited initiative. Its goal is to be a self-sustainable long-term service that will become an important part of the Australian dementia research infrastructure. StepUp has attracted a great deal of interest and support from individuals, communities, organizations, government, as well as dementia researchers across Australia, and has demonstrated its feasibility and value with good progress made during the early implementation phase. Strategic and concerted efforts are needed to address disparities in dementia research participation in StepUp and beyond. The groundwork has already begun and StepUp is poised to take the next step towards enhancing the platform and service to address issues of inclusion and diversity and to further improve user satisfaction and expectation. Fast tracking dementia research is a global interest, strongly echoed by the work of World Dementia Council. International collaboration and partnerships between the existing recruitment registries are needed to build a new set of evidence around their social and economic impact and sustainability.

## Figures and Tables

**Table 1:** Case analysis of studies registered in StepUp for Dementia Research.

Design	Study Description	Sites	Mat’d (Scr’d)	Ineligible/ Declined	Enrolled Numbers	Duration (Days)	Overall Numbers
**Online survey** **(*n* = 14)**	* Research volunteers’ motivation, needs, wellbeing and experiences	Nationwide	692 (N/A)	N/A	301	N/A	301
	* The role of habit in maintaining physical activity	Nationwide	229 (N/A)	N/A	60	N/A	126
	* Healthy ageing, memory and technology	Nationwide	757 (N/A)	N/A	170	N/A	254
	* Impact of COVID-19 on wellbeing and access to support	Nationwide	264 (N/A)	N/A	71	N/A	137
	* Dementia, stigma and factors that affect self-disclosure	Nationwide	802 (N/A)	N/A	182	N/A	304
	* Impact of COVID-19 on people with dementia/carers	Nationwide	272 (N/A)	N/A	16	N/A	46
	* Needs assessment of technology in aged care	Nationwide	590 (N/A)	N/A	63	N/A	188
	* Quality use of medicines in people with dementia	Nationwide	866 (N/A)	N/A	96	N/A	151
	* The evidence-practice gap in young onset dementia care	Nationwide	275 (N/A)	N/A	N/A	N/A	214
	* Beliefs and attitudes towards dementia	Nationwide	1085 (N/A)	N/A	165	N/A	185
	Online healthy brain ageing psychoeducation and cognitive training	Nationwide	113 (N/A)	N/A	In progress	N/A	In progress
	Experiences of adult children carer for parent with dementia	Nationwide	252 (N/A)	N/A	In progress	N/A	
	Benefits of gardens and plants to caregiver’s wellbeing	Nationwide	252 (N/A)	N/A	In progress	N/A	In progress
	Use of therapy-informed music in dementia care	Nationwide	369 (N/A)	N/A	In progress	N/A	In progress
**Observational study (*n* = 17)**	* Carers’ experiences of medication advice for people with dementia	Nationwide	182 (155)	65/1	22	15	30
	* Changed behaviors with memory and cognitive decline	Nationwide	311 (60)	6/3	51	61	52
	* Impact of COVID-19 on the lives of people with dementia and carers	Nationwide	214 (N/A)	0/0	16	N/A	18
	* Patient decision aid for deprescribing cholinesterase inhibitors	Nationwide	333 (7)	0/0	6	99	17
	* Carer’s experiences during COVID-19	Nationwide	196 (Uk)	0/0	29	Uk	29
	Computerized cognitive tests	NSW	158 (17)	0/1	In progress	52	In progress
	Early biological markers in familial non-AD	Nationwide	346 (342)	90/11	20	215	In progress
	Financial skills in ageing and cognitive impairment	Nationwide	766 (42)	15/18	6	79	In progress
	Gait and cognition study	NSW	55 (0)	0/0	0	In progress	In progress
	Making an advance research directive	Nationwide	85 (10)	3/0	5	17	In progress
	Validation of the Montreal Cognitive Assessment	NSW	103 (0)	0/0	0	In progress	In progress
	Social health and reserve in people with dementia	Nationwide	34 (28)	17/5	1	13	In progress
	Effect of hearing loss on cognitive impairment	NSW	124 (123)	45/27	12	205	In progress
	Optic nerve decline and cognitive change	NSW	275 (260)	153/2	2	2	In progress
	Habits on physical activity in people with MCI or memory concern	NSW	33 (29)	13/10	0	In progress	In progress
	Safety for people with memory problems or dementia	Nationwide	65 (0)	0/0	0	In progress	In progress
	Benefits of physical activity and brain health	Nationwide	155 (0)	0/0	0	In progress	In progress
**Experimental study (*n* = 9)**	* Exercise intervention in people with MCI (RCT)	NSW	45 (45)	41/4	0	N/A	68
	** Effects of intranasal oxytocin on emotions	NSW	14 (1)	0/0	0	N/A	5
	Sailuotong for VD or AD mixed with CVD	NSW, SA	23 (12)	3/4	1	262	In progress
	Melatonin supplementation in MCI (RCT)	NSW	19 (19)	10/6	2	119	In progress
	Online training and support for people with early-stage dementia and carers	NSW, QLD, VIC	118 (57)	0/21	1	17	In progress
	Driving cessation and mobility in people with dementia (RCT)	NSW, QLD, VIC, WA	364 (24)	3/13	3	198	In progress
	Telehealth psychotherapy for anxiety in people with cognitive impairment	Nationwide	105 (50)	4/7	1	71	In progress
	Music and reading for people with Dementia (RCT)	Nationwide	382 (327)	68/24	25	175	In progress
	Polypharmacy and reduction for general practice patients with dementia	WA	36 (35)	18/10	6	138	In progress

* Studies closed/completed; ** Studies closed/terminated due to external issues, a drug supply, geographical location, and the COVID-19 pandemic; Mat’d (Scr’d), Matched (Screened); NSW, New South Wales; QLD, Queensland; SA, South Australia; VIC, Victoria; WA, Western Australia; N/A, not applicable; Uk, Unknown; AD, Alzheimer’s disease; VD, Vascular dementia; MCI, mild cognitive impairment; CVD, cerebrovascular disease; RCT, randomized controlled trial.

**Table 2:** Survey participants’ demographics.

Characteristics		2019 Survey (*n* = 319)	2021 Survey (*n* = 450)
Gender	Female	80%	78%
	Male	20%	22%
Age	18–44	8%	9%
	45–64	42%	40%
	65+	51%	51%
Country of birth	Australia	67%	70%
	Other (UK, NZ, etc.)	33%	30%
Preferred language	English	99%	99%
Education	Year 11 or below	12%	11%
	Year 12 or Diploma	24%	26%
	Bachelor’s Degree	28%	27%
	Graduate Diploma/Certificate	13%	11%
	Postgraduate Degree	23%	26%
Marital Status	Single	9%	12%
	Married/in de-facto	66%	67%
	Separated or divorced	14%	11%
	Widowed	10%	9%
	Unwilling to answer	1%	1%
Employment Status *	Full-time	18%	21%
	Part-time	24%	20%
	Student	4%	2%
	Home duties/carer	5%	6%
	Retired	51%	47%
	Unemployed	1%	2%
	Pension/Allowance	11%	12%

* Represents questions allowed multiple choices.

## Data Availability

The datasets generated and analyzed during the current study will not be publicly available due to privacy and confidentiality reasons, but survey data may be available from the corresponding author upon reasonable request.
